# Evaluating the outcomes and implementation determinants of interventions co-developed using human-centered design to promote healthy eating in restaurants: an application of the consolidated framework for implementation research

**DOI:** 10.3389/fpubh.2023.1150790

**Published:** 2023-05-18

**Authors:** Melissa Fuster, Emily Dimond, Margaret A. Handley, Donald Rose, Charles Stoecker, Megan Knapp, Brian Elbel, Cara Conaboy, Terry T. K. Huang

**Affiliations:** ^1^Department of Social, Behavioral, and Population Sciences, Tulane University School of Public Health and Tropical Medicine, New Orleans, LA, United States; ^2^Partnership for Research in Implementation Science for Equity (PRIDE) Center and Department of Epidemiology and Biostatistics, School of Medicine, University of California, San Francisco, CA, United States; ^3^Department of Health Policy and Management, New Orleans, LA, United States; ^4^Department of Public Health Sciences, Xavier University of Louisiana, New Orleans, LA, United States; ^5^Department of Medicine, New York University Grossman School of Medicine,New York, NY, United States,; ^6^Wagner Graduate School of Public Service, New York University,New York, NY, United States; ^7^Center for Systems and Community Design and NYU-CUNY Prevention Research Center, City University of New York Graduate School of Public Health and Health Policy, New York, NY, United States

**Keywords:** restaurant, nutrition, human-centered design, consolidated framework for implementation research, implementation science, Hispanic (demographic)

## Abstract

**Background:**

Restaurants are an emerging yet underutilized setting to facilitate healthier eating, particularly among minoritized communities that disproportionately experience health inequities. The present study aimed to examine outcomes from interventions co-developed using Human-Centered Design (HCD) in two Latin American restaurants, including sales of healthier menu items (HMI) and the consumer nutrition environment. In addition, we aimed to assess implementation outcomes (acceptability, fidelity, and sustainability) and elucidate the determinants for implementation using the Consolidated Framework for Implementation Research.

**Methods:**

This study used a mixed-methods, longitudinal design. Data were collected pre-, during, and post-intervention testing. Intervention outcomes were examined through daily sales data and the Nutrition Environment Measures Survey for Restaurants (NEMS-R). Changes in HMI sales were analyzed using interrupted time series. Implementation outcomes and determinants were assessed through site visits [observations, interviews with staff (*n* = 19) and customers (*n* = 31)], social media monitoring, and post-implementation key informant interviews with owners and staff. Qualitative data were analyzed iteratively by two independent researchers using codes developed *a priori* based on CFIR.

**Results:**

The HCD-tailored interventions had different outcomes. In restaurant one (R1), where new HMI were introduced, we found an increase in HMI sales and improvements in NEMS-R scores. In restaurant two, where existing HMI were promoted, we found no significant changes in HMI sales and NEMS-R scores. Acceptance was high among customers and staff, but fidelity and sustainability differed by restaurant (high in R1, low in R2). Barriers and facilitators for implementation were found across all CFIR constructs, varying by restaurant and intervention. Most relevant constructs were found in the inner setting (restaurant structure, implementation climate), individual characteristics, and process (HCD application). The influence of outer setting constructs (policy, peer pressure) was limited due to lack of awareness.

**Conclusion:**

Our findings provide insights for interventions developed in challenging and constantly changing settings, as in the case of restaurants. This research expands the application of CFIR to complex and dynamic community-based settings and interventions developed using HCD. This is a significant innovation for the field of public health nutrition and informs future interventions in similarly dynamic and understudied settings.

## Introduction

Eating out is increasingly common today. The consumption of foods prepared away from home accounts for 50% of food spending among American households (1). This is important, as restaurant foods are associated with increased intakes of saturated fat and sodium, increasing risks for diet-related diseases, such as diabetes and cardiovascular disease (2–4). Cardiovascular disease is the leading cause of death in the United States (US), where about 7.2% of the population has been diagnosed with coronary heart disease (5). Diabetes is more prevalent, affecting 11.3%, plus 38% of US adults have pre-diabetes (6). At the same time, restaurants can serve as vehicles to spread culinary innovations by exposing consumers to new ingredients and preparations and changing social norms to motivate healthful eating practices, potentially affecting the foods cooked at home (7, 8). Public health initiatives and policies to improve food choices at restaurants have included efforts to restrict choice (e.g., trans-fat ban law) or guide choice through pricing schemes, point-of-sale promotion of healthy options, and nutrition information (9, 10). Research has also documented voluntary changes made by the industry to promote healthier choices (10, 11). However, most of these efforts have targeted and focused on chain-based, fast-food restaurants. While this focus is important, it fails to engage independently-owned, non-chain restaurants, which make up more than half (53%) of the industry in the United States (12). Emerging research in independently-owned restaurants demonstrates interventions can be successful at increasing the consumption of healthier options, through point-of-purchase promotion of healthy dishes and increasing the availability of healthier options (13). However, these efforts tend to exclude non-chain, minority-serving restaurants (9, 13). This is a missed opportunity to engage the sector for culinary innovations that may promote healthier diets and enhance equitable access to healthy foods among communities at greatest risk for diet-related conditions, as in the case of Latin American communities in the US (14–16). According to the National Restaurant Association, 80% of consumers eat at a restaurant serving ethnic cuisine at least once a month (17). Within these, there are over 120,000 Latin American restaurants in the US, most of which are independently owned. Mexican restaurants alone make up 8% of all US restaurants (18, 19). Yet, despite their importance, Latin American restaurants (along with other ethnic restaurants) remain an understudied and under-engaged sector. This research addresses this gap by applying implementation science to understand factors influencing restaurant engagement in community nutrition interventions. Restaurants are promising settings for interventions, but the lack of understanding of organizational context and determinants for implementation limit the capability of these programs and the dissemination for sectors in greatest need, as in the case of restaurants serving Latin communities.

## Materials and methods

### Study overview

This study examined the intervention and implementation outcomes of pilot initiatives developed using Human-Centered Design (HCD) approaches in two Latin American restaurants located in New York City (referred to as R1 and R2). HCD is an approach to developing solutions rooted in an experimental process and the needs and context of the end user to develop bottom-up solutions. The process has been increasingly used in public health interventions (20, 21) and is suitable for working with restaurants, given the unique circumstances and barriers affecting these establishments, particularly independently-owned restaurants. Given the importance of user-centeredness, it is expected that interventions co-developed with end users through this approach should result in greater acceptability, fidelity, and sustainability (20, 22). The present study aimed to (1) examine the effect of the resulting interventions on the sales of healthier menu items (HMI) and the consumer nutrition environment, (2) assess implementation outcomes (acceptability, fidelity, and sustainability) (23), and (3) elucidate the determinants for implementation using the Consolidated Framework for Implementation Research (24), a widely used determinant framework in implementation sciences to examine the intricacies of complex settings examining implementation as a social process that is interwoven with the context in which it takes place (25). The framework has been primarily applied in healthcare settings (26), with few community-based applications, even less in restaurants (27, 28).

### Restaurant recruitment and overview

We worked with two Latin American restaurants located in New York City. The restaurants were identified through an ongoing community-engaged process, starting with listening sessions with Latin American restaurants beginning in October 2020 to examine barriers and facilitators for engaging in healthy eating promotion strategies (29). Restaurants were initially recruited through social media outreach and community networks, including a snowball approach. From the listening sessions, we identified an initial group of five restaurants that expressed interest in collaborating with the project. These restaurants were all located in New York City, given the team location at the time. They included three full-service and two counter-style restaurants. Three restaurants dropped out in response to issues related to the business, including loss of staff and temporary closures related to COVID-19. The two participating restaurants were a counter-style restaurant serving Puerto Rican food in a food hall (R1) and a full-service Mexican restaurant (R2). The participating restaurants received $300 as a stipend for participation, plus reimbursement for key intervention costs (i.e., new menu board in R1 and cost for photography in R2). Additional incentives included restaurant promotions on project social media and incentives ($50 gift card) provided to individuals (owners and staff) for participating in the data collection efforts.

### Intervention description

We engaged owners and staff throughout the intervention design process, including problem definition, solution ideation, and the testing and refining of potential solutions, following the Stanford d. School HCD process (30, 31). Our iterative process is detailed in a separate publication (in process). Briefly, we engaged owners and chefs through one 3 h workshop where we defined the problem to be addressed and potential solutions. This was followed up by subsequent 1 h meetings where we refined potential solutions briefly tested (prototyped) by the partner restaurants. The workshops were co-facilitated by a designer and the study lead investigator. The process resulted in two tailored interventions to promote healthier choices, based on the needs identified by the restaurant stakeholders. In R1, our research and discussion with the restaurant stakeholders elucidated the need to increase healthier offerings in the menu that were also acceptable and profitable. The restaurant was offering a green salad that was underselling, resulting in food waste and lost profits. The chef developed a new offering, the *verduras*, a seasonal mix of non-starchy vegetables (cabbage, squash, peppers) seasoned with traditional spices. The verduras were added to multiple dishes in the menu, and the menu was re-designed accordingly. We also added avocado slices as a healthy side alternative. Our research in R2 led to a different identified problem and solution, where the menu was already offering innovative, healthier options, but these were not being promoted or seen as culturally authentic by some customers. We worked with the owner to develop social media messaging to promote these offerings by touting their sensorial characteristics (e.g., taste, texture) and connection with tradition and history, to dispel customer misconceptions of the cuisine being unhealthy and the healthy offerings not being part of the Latin American cuisine (i.e., lack of authenticity).

### Data collection procedures and participants

**Figure 1:**
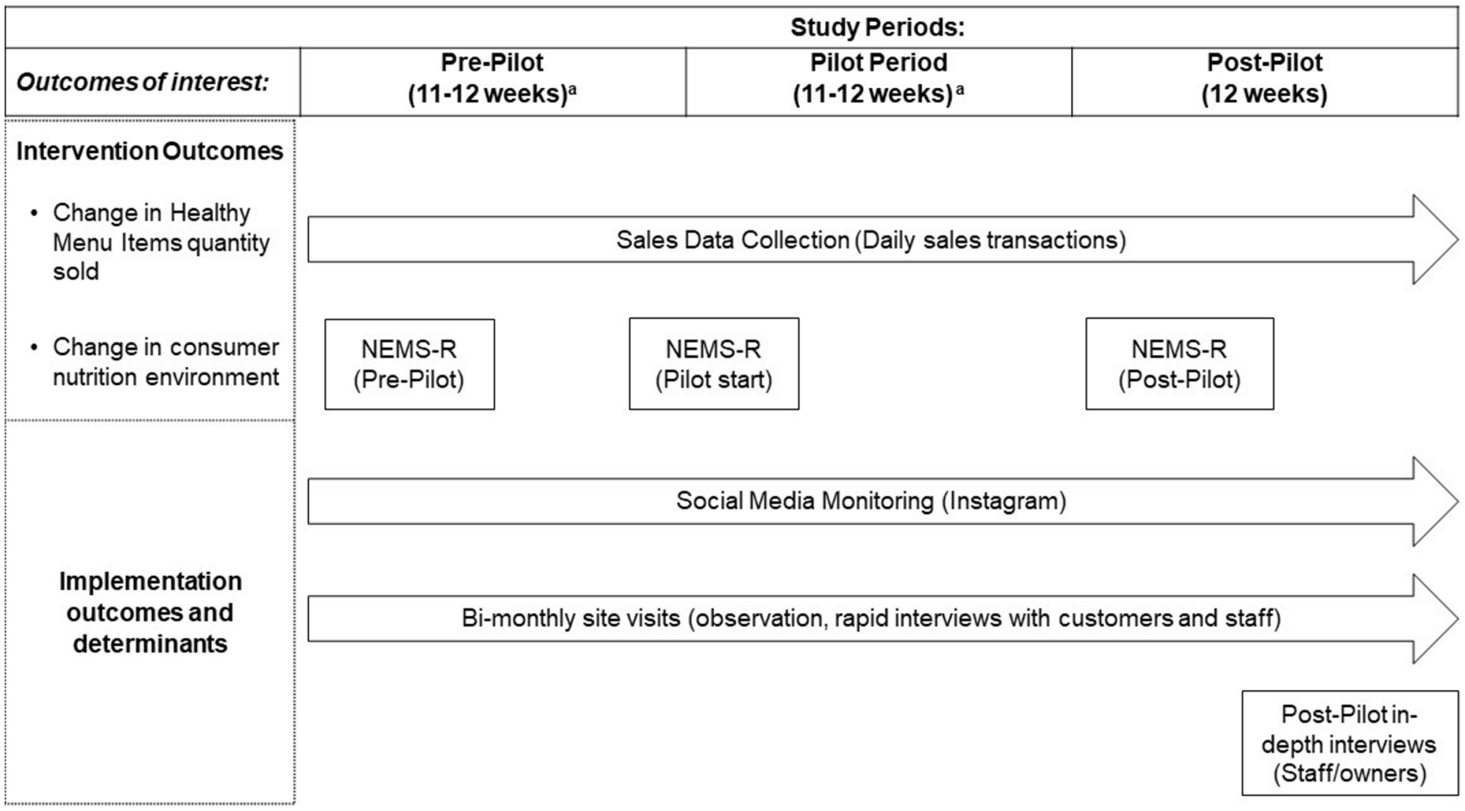
Data collection efforts by study period and outcome of interest. NEMS-R, Nutrition Environment Measures Survey for Restaurants. Social media monitoring also used to examine change in consumer nutrition environment. ^a^Pre and implementation period for R1 was 11 weeks to accommodate owner’s preferences related to new menu roll-out and revisions.

This study used a mixed-method approach to examine and link intervention and implementation outcomes. The study combined data collected across the implementation process (pre, during, and post), incorporating sales data, ongoing rapid interviews with staff and customers, guided environmental observations, and in-depth key informant interviews conducted with owners and staff members at the conclusion of the study (Figure 1).

#### Intervention outcomes assessments

We assessed change in HMI sold using sales data collected *via* the partner restaurants’ point of sales (POS) systems (Toast, Boston, MA; Breadcrumb, Providence, RI), which are computerized systems that allow restaurants to track and manage onsite and online orders (including from third-party sites), used as part of restaurant financial management. We first identified HMI in collaboration with a registered dietitian with expertise in Latin American diets. We considered three criteria: ingredients, preparation, and level of culinary innovation (Table 1). The criteria were not based on quantified nutritional benchmarks, allowing for some level of flexibility and taking into account if the offering was healthier in comparison with the usual alternatives found in similar restaurants. In R1, HMI were mainly those that incorporated the new vegetable component (verduras), which was considered an innovation. This included using verduras as a side, base for bowls (as opposed to rice) or in place of meat, but we also counted a leafy green side salad that was available before the start of the intervention. In R2, examples of existing HMI included a cabbage salad, fluke ceviche tostadas, roasted cauliflower tacos, and a vegetarian sandwich (torta), among others fitting the criteria (Table 1). Data were downloaded from the restaurant POS system as individual transactions covering our study periods (Figure 1). Individual transaction data were collapsed to the day level, including totals for item sales indicators. Given that the data are used for sales, data on individual customer consumption (including table size) are not tracked in a reliable manner. Therefore, we could not examine HMI as *per capita* daily sales.

**Table 1:** Healthier menu item (HMI) criteria.

Criteria	Definition and examples
Ingredients	Item contains ingredients that are nutrient-rich with known health benefits, such as avocado, fish/seafood, fruits and vegetables, and item does not contain a high proportion of ingredients that are high in fat (e.g., cream, cheese) and simple carbohydrates (e.g., white rice^a^).
Preparation	Offerings are not fried or cooked with added fats, or fried component is not the main component of an offering containing otherwise healthier or innovative ingredients.
Innovativeness	Offering is an innovation from usual offerings in similar restaurants. Examples include plant-based substitutions for traditional meat-based dishes, seafood substitutions for traditional beef/pork-based dishes or vegetarian offerings.

a Exception made for items in R1 that contained verduras (the new vegetable-based offering), for example, shrimp over rice with a side of verduras.

We examined changes in consumer nutrition environments [ie. The environment experienced by consumers within restaurants (32)] using the Nutrition Environment Measures Survey for Restaurants (NEMS-R), a validated tool developed for this purpose, which examines food availability, and barriers and facilitators for healthier food choices in restaurants (33). The NEMS-R was applied at pre-, during and post-intervention, with a trained team member carrying out guided observations and menu assessments. We modified the protocol to assess the proportion of menu items classified as HMI. Following the NEMS-R protocol, we focused on main dishes (entrees), assessing side dishes separately for the availability on non-starchy, non-fried sides. We expanded the NEMS-R assessment of promotional efforts to examine those undertaken on social media. This was done given the emphasis on social media promotion in R2 and the increased use of social media for promotion efforts by restaurants. Our assessment focused on Instagram as the primary platform used by restaurants in general, as confirmed by our partner restaurants and in our formative data collection efforts (29). We collected social media posts in a database (AirTable), including the image(s) posted and captions throughout the study period (Figure 1).

#### Implementation outcomes and determinants assessment

Our examination of implementation outcomes and determinants was guided by Proctor et al.’s (23) implementation outcomes framework and the CFIR framework, using site visits and semi-structured interviews with owners and staff (Figure 1). Given that the intervention for R2 was focused on social media promotion, we used our social media monitoring effort to track fidelity and sustainability as well.

Site visits were carried out throughout the duration of the study (Figure 1), including one visit in the pre-test period, and planned bi-monthly visits in the testing and post-testing periods. We conducted a total of 14 site visits. R1 received three visits during the testing period and three during the post-testing period. R2 received four during the testing period and two during the post-test, with a third visit canceled due to the uptake in COVID-19 infections that coincided with the post testing period in January 2022. The site visits included a check-in with restaurant owners, short, structured interviews with staff and customers, and observations of the restaurant environment. Each site visit included a quick check-in with the owner and short structured interviews with 1–2 staff members, depending on availability during the day of the visit (total interviews = 19; 9 in R1 and 10 in R2). The staff included both front of the house (servers, cashiers) and back of the house (chef, cooks). The short interviews were on site (e.g., at the kitchen, by the cashier), based on interviewee preference and to be as unobtrusive as possible. The staff interviews included questions about intervention awareness (first encounter only), opinion of the intervention, perceived changes in customer ordering of healthier options, and whether partnering with the project changed their work. These conversations were short, lasting around 10–15 min, depending on staff availability and how busy the restaurant was on the day of the visit.

Customer perspectives were captured through short intercept interviews with customers present at the day of the site visit, a method commonly used in food retail intervention studies (34). A trained team member approached customers after ordering or while food was consumed for a short, structured interview to assess customer satisfaction with offerings and perceptions of the intervention-related outcomes, including opinions concerning healthy offerings at the restaurant and in Latin American restaurants in general. A standard set of questions was followed to ascertain how frequently the customer ate at the restaurant, what they ordered, if they tried any of the healthier options, and the reason behind their choice. After a brief explanation of the project, they were also asked if they thought the project was a good fit for the restaurant and what other health-focused initiatives they would like to see. We conducted an average of 3.5 customer interviews per site visit in R1 (21 total) and 2.2 interviews per site visit in R2 (10 total). The customers interviewed ranged in age from their 20s to 50s, with the majority being young adults in their 20s to early 30s. We had a close to even split by gender (male/female). The majority of these customers were Latin/Hispanic or Non-Hispanic White. The Latin background of most of the Hispanic customers coincided with the restaurant cuisine served.

The site visits also included non-participant observations to note overall patron volume, demographic characteristics, patron-patron interactions, patron-staff interactions, food orders, and factors facilitating/inhibiting healthy item ordering. Interactions were observed during orders to assess customer interactions with the menu, questions about offerings, and if HMIs were being discussed or promoted. The factors observed related to HMI orders were mostly environmental ones, including which products were showcased at the point of sale and potential promotions offered on site.

After the conclusion of the post-pilot period, we conducted in-depth interviews with restaurant owners and staff, including one front-of-the house staff (server, cashier) and one back-of-the-house staff (lead chef). Staff were made aware that their participation was voluntary and that neither their personal information nor information provided in the interview would be shared with their employer. Participants received $50 as compensation for their time. The interviews lasted, on average, 37.2 min. The interview guide was based on the CFIR interview guide (24), covering the framework domains: intervention characteristics, which encompass attributes of the intervention that influence the success of the implementation, including whether the intervention was perceived as internally or externally developed, complexity and required level of organizational reorientation, and costs; the outer setting, encompassing peer or competitive pressure to implement an intervention, the importance of client needs, connectedness with other organizations, and the influence of external policies and incentives; the inner setting, referring to the social architecture of the organization, available resources, culture, implementation climate, tension for change, and compatibility of the intervention and the organization, among other related factors; characteristics of individuals involved, including knowledge and beliefs about the intervention, individual belief in own capabilities, readiness of change, and other personal attributes (tolerance for ambiguity, innovativeness, etc.); and the process by which the intervention is implemented (25) (Table 2). The interviews were conducted *via* Zoom by two trained, bilingual interviewers -one lead interviewer and one co-facilitator/note-taker. The team debriefed after each interview, discussing insights gained, which were then shared during team study meetings in preparation for analysis.

**Table 2:** Interview sample questions by CFIR domain.

CIFR Domain	Selected question examples
Intervention characteristics	Do you think the changes were difficult to implement? Did the changes make your work very different from how it was done before? What were the costs to making the changes? How were the changes developed? Who developed the changes? Inclusion in process?
Inner setting	How did the characteristics or set-up of [Restaurant] affect the implementation of the changes? Why were the changes developed at [Restaurant]? Do you think there was a strong need for this change? How well do you think the changes and the collaboration fit with the values, mission or norms within [Restaurant]?
Outer setting	How well do you think the intervention met the needs and wants of [Restaurant’s] clients? Are there barriers for clients to benefit from the changes promoted by the intervention? Can you tell me what you know about any restaurants similar to [Restaurant] that are doing similar innovations? Were there financial incentives or other incentives that influenced your decision to participate? Are you aware of regulations, policies or guidelines at the national, state, or local levels that may promote healthier eating in restaurants?
Process	Crosscutting domain, capturing reflections and evaluations across the different areas
Characteristics of individuals	How would you describe the changes or innovations implemented? (knowledge and attitudes of interviewees)

The procedures involving human participants were reviewed and deemed as exempt by Tulane University School of Public Health and Tropical Medicine and the City University of New York. The participants provided their verbal informed consent to participate in this study. Written consent was not required as the research presented no more than minimal risks and the written consent would be the only record linking the subject and the research.

### Data analysis

#### Changes in HMI items sold

The main intervention outcome was the number of HMI sold per day. Preliminary analyses of sales data were shared and discussed with owners to discuss emerging trends and to serve as part of our ongoing engagement with the partner restaurants, informing decisions concerning our analysis approach of focusing on HMI as quantity sold, as opposed to using dollar amount sold. Interrupted time series analysis was used to examine trends and breaks in trends in daily sales of HMI across the three study periods (Table 1). Analyses were conducted using the STATA BE 17 “itsa” command (35). Days when a restaurant was closed were treated as missing. Statistical significance was established at *p* < 0.05.

#### Changes in consumer nutrition environments

Changes in the consumer nutrition environments were done based on NEMS-R factors, including a mix of dichotomous indicators (Yes/No) and menu proportion calculations to examine the proportion of menu items classified as HMI across the three study periods. We adapted NEMS-R protocol to calculate the resulting NEMS-R scores (33), to assess differences by study period.

#### Analysis of social media posts

Social media posts (images and captions) were coded by two team members independently. After an initial pass, coders were reviewed by team members and during team meetings, where codes were clarified and reconciled, as necessary. The codes were simple and descriptive, noting if the post promoted a HMI (e.g., image of HMI included and/or caption promoted the item) or whether the post promoted unhealthy items or overeating (e.g., post featuring fried foods). Posts that did not feature food (e.g., event promotion, merchandize) or only featured alcohol were coded as non-food messaging.

#### Analysis of site visit data

Data from the site visits were entered into a database. Open responses from customers and staff interviews were summarized using descriptive, summative codes developed from responses (open coding) applied by the team member conducting the site visits, and subsequently revised and discussed with a second team member and the study PI. Notes from the site visit were also summarized, tracking time of visit, client volumes, and staff presence.

#### Qualitative analysis of post-test key informant interviews

The post-test semi-structured interviews were transcribed verbatim. Five interviews took place in English and one in Spanish, which was translated to English prior to analysis. The analysis used a directed content analysis approach (36), a deductive approach where codes were developed *a priori* using the CFIR framework interview development guide tool (24). The textual data were coded independently by two team members using NVIVO v.12 using an iterative approach that included ongoing coder debriefing and discussions, and larger meetings with the study PI to discuss emerging results. Excerpts were further organized according to the CFIR construct domain.

#### Data triangulation

Our work incorporated various data sources to understand the implementation of the tailored interventions. Data collected from the site visits were used to triangulate the information collected *via* the post-pilot key informant interviews. This validation was undertaken during debriefing meetings, where notes from the site visits were compared with findings emerging from the interviews. The site visits, key informant interviews, and social media analysis also helped contextualize the trends we observed in the HMI and NEMS-R analyses. These data were discussed during research team meeting, as well as during ongoing meetings with owners (during and post-testing), where we shared our emerging findings, including sharing detailed sales trends and our findings from the social media analysis.

## Results

### Intervention outcomes

#### Changes in sales

**Figure 2:**
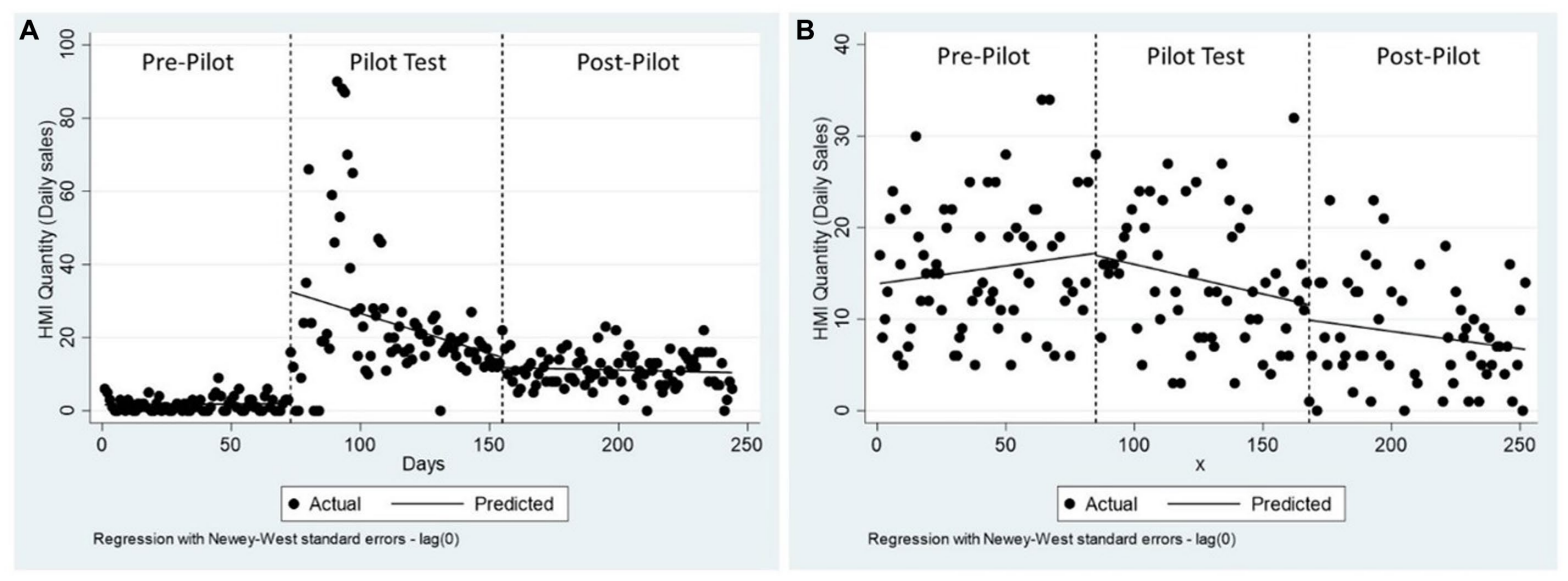
Changes in HMI daily items sold across study periods in R1 **(A)** and R2 **(B)**.

On average, overall the sales of HMI made up a small proportion of the value of food sales in dollars (3% of food sales in R1 and 22% of food sales in R2). On average, R1 sold 12.6 ± 14.3 HMI (8.7% of all food items sold, as individual items) and R2 sold 12.8 ± 17.4 HMI on a daily basis (21.8% of all food items sold, as individual items). In R1, the intervention resulted in an increase in HMI sales by 31 units, followed by a decrease in HMI sales of 0.22 unit per day. After the testing period ended, daily sales of HMI were not significantly different from baseline (Figure 2). In R2, the intervention did not have a significant influence on the quantity of HMI sold (Figure 2; see Supplementary Table S1 for regression results).

#### Changes in consumer nutrition environments

Table 3 presents an overview of key indicators in the restaurants’ consumer nutrition environments across the three study periods. R1 showed a lower proportion of HMI in menus at baseline, increasing from 15 to 53% as a result of the menu changes. However, R1 also showed fewer facilitators for the promotion of healthier choices, including the promotion of unhealthy items (i.e., fried snacks) in social media and the overall encouragement for overeating through large portions (Table 3). R2 presented more facilitators for healthier choices, including the availability of main dish salads, half-portions, and the promotion of healthier items in social media, the latter, as part of the intervention. In concordance with the intervention, R2 showed an increase in social media promotion of HMI between baseline and the intervention period, but a decrease after the pilot period, denoting that the intervention was not sustained (Table 3).

**Table 3:** Selected consumer nutrition environment indicators and total NEMS-R scores by restaurant and study period.

	R1: New HMI & Menu Redesign			R2: Social media promotion of HMI		
	Pre-Pilot	Pilot test	Post-Pilot	Pre-Pilot	Pilot test	Post-Pilot
NEMS-*R* Score	4	7	5	11	11	8
*Food availability*						
Whole grains	No	~	~	No	~	~
Fruit without added sugar	No	~	~	No	~	~
Nonfried, nonstarchy vegetable side	Yes	~	~	Yes	~	~
Main dish salad	No	~	~	Yes	~	~
Healthier menu items, *n* (%)	14 (15%)	73 (52.7%)	55 (47.8%)	7 (29.2%)	~	~
100% fruit juice	No	~	~	Yes	~	~
*Facilitators to healthy eating*						
Reduced/half portions of main dishes offered	No	~	~	Yes	~	~
Healthier options highlighted on site / menu	No	~	~	No	~	~
Healthier options promoted in social media (% of Instagram posts)*	2%	10%	15%	22%	48%	11%
Smaller portions cost less than regular ones	NA	~	~	Yes	~	~
*Barriers to healthy eating*						
Large portions encouraged	Yes	~	~	No	~	~
Unhealthy options highlighted on site / menu	Yes	~	~	No	~	~
Unhealthy food options promoted in social media (% of Instagram posts)*	56%	57%	48%	0%	~	~
Healthier items cost more than comparable, regular items	No	~	~	No	~	~

* Not part of NEMS-R Scoring; ~ denotes indicator was unchanged across periods.

### Implementation outcomes

#### Acceptability

Acceptability was overall high in both restaurants among customers, owners, and most staff. The staff rapid and in-depth post-test interviews revealed that most staff at both restaurants had a positive attitude toward the intervention and recognized its benefits.

They’re always talking about having good quality healthy food for affordable price. I think that's the idea of them like having good stuff, good quality stuff, stay healthy, and then have lower prices, it's good. It's good for their Instagram as well. - R2 server

The staff in both restaurants also confirmed that the intervention did not increase workload nor had any unintended consequences regarding decreased tips or revenue in both restaurants. An exception to this was the chef in R1, who expressed ongoing resistance through the intervention development process, resulting in low initial acceptance of the change. The low acceptance was associated with the perception that the intervention was not a good fit for the restaurant brand – a finding is further elucidated in the next section, as part of the CFIR analysis.

The customer intercept interviews conducted during the site visits showed that most customers at both businesses saw a need to eat healthier at restaurants, with a higher proportion of interviews showing this in R1 versus R2 (71.4% vs. 50%, respectively). Only one client at R2 expressed that it was not the role of restaurants to facilitate healthier eating. In R1, most rapid customer interviews (15 out of 21, 71.4%) saw the need for healthier eating, and some expressed the desire for fewer fried items (2 out of 21), saw healthy options as good for the business (4 out of 21), and one client noted that they would recommend the restaurant based on the availability of healthy items. Customer acceptability was also noted by staff in regard to the new offerings in R1.

I think they [customers] have taken to it, because customers now come and immediately order a side of vegetables or [the verdura] bowl. That’s how we know customers have taken to it in a good way, that they have accepted the project. - R1 Cashier

#### Fidelity

The site visits and social media monitoring were used to examine implementation fidelity. In R1, the visits confirmed that the new menu items were being continually kept throughout the testing period. In R2, fidelity was assessed as social media engagement, monitored *via* Instagram, as presented in Table 3. In R2, social media postings increased from a total of 9 (on average, 0.75/week) in the pre-testing period to 27 (2.25/week) in the testing period, with an increase in posts that formally showcased an HMI during the testing period (Table 3).

#### Sustainability

The intervention was largely sustained in R1, where the main intervention addition –the verduras –was kept on the menu. As shown in Table 3, there was a slight decrease in HMI available after the testing period. This was due to the restaurants taking out the avocado side added along with the verduras as part of the intervention after the conclusion of the testing period. This change responded to the high cost of the item, lack of reliable sourcing and quality, and difficulties in the preparation logistics. In R2, the social media postings decreased after the testing period, denoting a lack of sustainment of the innovation implemented.

### Determinants for implementation: application of the CFIR

**Figure 3:**
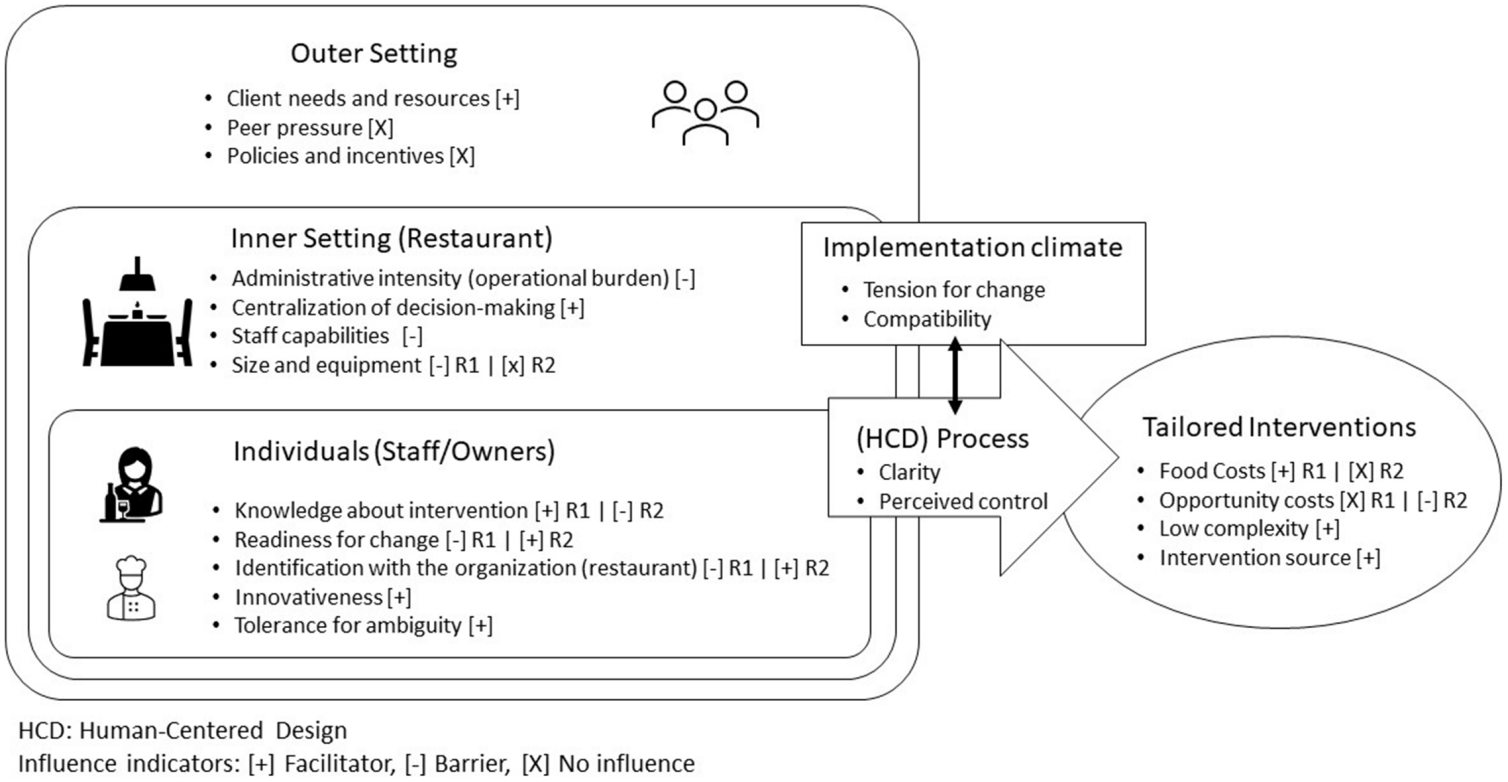
CIFR model for factors influencing the tailored intervention development and implementation.

Figure 3 summarizes our findings, illustrating relevant CFIR constructs within each domain as facilitators, barriers or factors with no perceived influence. This section is organized according to the type of influence, discussing the domains and constructs as interacting to either facilitate the changes or hinder intervention impact, ending with factors in the outer context that were perceived as having no influence.

#### Facilitators: what enabled the changes observed?

*The use of HCD resulted in high owner buy-in for the resulting interventions (process and intervention characteristics).*

Our use of HCD to engage restaurant owners and staff in the intervention development process yielded simple, restaurant-developed changes that were low in perceived costs and high in owner acceptance. The application of HCD resulted in changes that were internally developed, which served as a facilitator for implementation. The use of HCD increased the tension for change needed to facilitate intervention adoption, promoting changes within the inner context through interactions with the individuals involved (Figure 3). This was the case given the sharing of information and engagement of the restaurant stakeholders in the process, which provided an opportunity for reflection about potential business improvements,

At restaurants, you fall into [a] way [of how] you do things. There's never time to stop and reflect for too long. […] When you have an outside entity that can come in and point out certain things and [make changes doable]. It's been very helpful. -R2 Owner

In both restaurants, the intervention was perceived as relatively simple, with low costs and requiring minimal reorientation. In R1, the owner said that the intervention was beneficial given the lower cost of the ingredients used in the new vegetable offering, which he perceived to be very popular.

Money-wise, it has been a success because it cost me less for the kinds of vegetables that we're using and we're selling more of them because they became very popular. - R1 Owner

R2’s owner found the content created for social media posts about the health benefits of specific ingredients, connections to the cuisine’s origin, and the professional photographs beneficial for promotion, facilitating the implementation of the changes.

*Owner’s innovativeness and tolerance for ambiguity facilitated buy-in and implementation (characteristics of individuals).*

Innovativeness and tolerance for ambiguity were key characteristics of the owners, facilitating their desire to be a part of the intervention, even though this was a new experience with unknown results, as shown in this excerpt:

I guess the biggest challenge for anyone that's going to go through something like this is [to] give a chance [for the intervention] to work because if you're quick to judge, it's not going to work out. I'm not quick to judge and I let it go. -R1 Owner

When first approached, the owners were contemplating changes. This sentiment may be partly associated with the context in which the engagement began, in the midst of COVID-19, a period when restaurants were forced to adapt to an ongoing climate of uncertainty. Owners primarily expressed this tolerance of ambiguity, more so than chefs or front-of-house staff, who were constantly reorienting their work according to the changing climate during COVID-19.

Owners are the primary decision-makers in the restaurant and are responsible for future initiatives and the business’s trajectory. These two traits facilitated the engagement of these restaurants with the use of HCD approaches, where the intervention emerged from the engagement process, as opposed to restaurants being prescribed specific changes. This increased the acceptance of the resulting intervention but required patience for the creation of tailored approaches. For instance, R1 owner noted the lack of clarity in the pre-intervention period, where the end product of the HCD process was not clearly specified –an ambiguity that is part of the process.

*Owners perceived the intervention met client needs and presented an opportunity to expand the customer base (Outer Setting, individual characteristics).*

The influence of client needs was an important facilitator for restaurant engagement and intervention adoption, influencing readiness for change and suggesting interactions between the outer setting and individual characteristics (Figure 3). Both owners recognized a growing demand for healthier offerings, including vegetarian offerings, among clients in general. In R1, the addition of new dishes that incorporated verduras was seen as a positive change to accommodate more customers. The owner of R2 thought that his restaurant’s vegetable-focused offerings highlighted in the social media intervention would cater to the majority of the restaurant’s current customer base, consisting of a young, white clientele interested in vegetarian and vegan offerings. However, he was also concerned with reaching more community members where the restaurant was located, many of whom were Hispanic, a need that was addressed *via* social media messaging, connecting the restaurant to the community and the contemporary cuisine in Mexico.

*Centralized decision-making moved intervention forward, despite staff resistance (inner setting, individual characteristics).*

The participating restaurants were independently owned and small in size, resulting in a centralized decision-making process. This was key in moving the intervention development and implementation process forward, especially in R1, where we found resistance and different perspectives coming from the chef.

I was a little bit against a lot of the changes as you know. So I think that a lot of the seed was planted by you guys and then ultimately that was [the owner] over my head that did all the changes […] I didn't necessarily have a say in it at the time, but as far as I know, you guys were on the one side, pushing [the owner] into that idea of having those vegetables. That's something that we talked about, but sometimes you need to hear from a third party in order to light a fire under his a** if you want to do something. - R1 Chef

Several characteristics of individuals, including knowledge about the intervention and identification with the organization, relate to the centralized decision-making (inner setting, Figure 2). Knowledge was high among owners and chefs, who were part of the intervention development process and also had managerial positions within the restaurants. Individual identification refers to how individuals perceive the organization, and their relationship and degree of commitment with that organization. Respondents showed strong identification with the restaurants, with both owners and staff showing a high level of commitment the success of the restaurant. For owners, the identification was more personal, as the restaurants were essential to their livelihood and the result of their personal vision. Chefs and front-of-house staff, on the other hand, may change employment without losing a critical financial investment. Ultimately, this difference contributed to centralized decision-making by the owners regarding the intervention.

*Partner restaurants had the resources to create and implement the intervention (inner setting, intervention characteristics).*

Competence was important for creating and implementing the changes. Culinary skills were important for R1 chef to develop the verdura offerings, and then train the staff to sustain the dishes. The new dishes were internally developed, based on existing resources and capacities, which were aspects that supported implementation and sustainability. In R2, the owner had the skills and knowledge to engage in social media promotion for the restaurant. Our collaboration augmented the existing skills by providing additional resources for crafting the messages and supporting the cost of additional photography. However, the burden of implementation was minimized by the pre-existing resources, including the existing social media accounts and established presence, as well as the existing HMI that were promoted.

#### Barriers: What hindered intervention-related changes and outcomes?

*Overall low tension for change limited the extent of the intervention (inner setting and process).*

While the HCD process facilitated owner buy-in and ultimate implementation of positive changes, the resulting intervention was not enough to create large impacts in HMI sales, especially in R2. As discussed by R2 chef, the social media intervention might not be the most effective way to reach the Latin communities in the area (mostly Puerto Ricans and Dominicans), who might be less familiar with the contemporary Mexican cuisine served in R2. Our interviews also showed that, in general, front of the house staff had low knowledge about the intervention, which was explained by the owner as being due to other responsibilities and time constraints. We sought to address this by extending the intervention to include staff training for promotion of HMI in-house, but this idea was not realized due to the owner’s time constraints and competing priorities.

*Staff capacity, COVID-19, and structural factors hindered expansion and effectiveness (individual characteristics, intervention characteristics, inner setting, outer setting).*

Restaurant staff capability and structural constraints were mentioned in both restaurants as limitations. In R1, both the owner and chef cited the small restaurant space as a barrier for change. R1’s owner spoke of how it is difficult to keep up with growing business in a small space, making it difficult to make changes. R1 chef mentioned several specific limitations, including staff size, kitchen layout, and capacity to change the menu.

It was just too many, I felt, limitations. Like I said, based on staff size, based on refrigeration, based on storage, based on everything. We're in a food hall. We can only do so much. We knew what we'd have to have on the menu already. We have to have this, we have to have this, these are the foundations of the restaurant, of the menu. There really wasn't a lot of room for wiggling around and adding a bunch of stuff. - R1 Chef

Both restaurants discussed facing issues with staff retention and turnover, requiring ongoing training and adding to the administrative burden. This was especially the case given the effect of COVID-19 and subsequent issues with hiring staff in general. In R2, staffing issues led to closures in the post-testing period, including short closures during the holidays and a reduction in the number of days the restaurant stayed open. This was also reflected in the diminished number of social media postings in the post-testing period. Another structural factor of importance for R2 was the layout, where the restaurant had a bar at the entrance, where part of the clientele came in mostly for drinks and not necessarily food, as noted by the chef,

I think that [the] biggest struggle of trying to [promote heathier eating] and grab other people's attention and not just be this boozy hangout. Even, it is set up that way. When you walk into the restaurant, the first thing you see is a big a** bar, and maybe that doesn't necessarily- people just walking by with their families think like, "Oh, that's a place I would like to have dinner." There were subtle changes made that have improved that aspect of the restaurant, but I think that was probably one of the biggest hurdles in a way.” - LL_Chef

Similar to R1’s chef, R2’s owner also mentioned restaurant size as a barrier to change because of staff size and kitchen layout.

*While interventions were seen as simple, costs and operational burdens prevented implementation intensity (inner setting and intervention characteristics).*

Administrative intensity, a construct from the inner setting domain and a structural characteristic, was particularly salient in both restaurants, especially given the small size of the staff, where owners had to take on multiple roles alongside running the restaurant. In R1, our engagement resulted in a simple intervention that required an initial level or reorientation, but then became “second nature.” While the intervention added an item to the menu, the costs were perceived as low, except for one item – the avocado – which carried higher cost and less reliability, resulting in it being taken off the menu after the testing period.

In R2, the lack of changes to the menu or the restaurant environment made the intervention initially simpler, compared with R1. However, the reorientation process was a more ongoing process, given the constant need for social media posting, a task that fell under the owner’s responsibilities, on top of the other tasks involved in running the restaurant. This resulted in a lower implementation intensity, given a low number of social media posts, as noted by the owner,

It was difficult, sometimes, to keep up with my part of it, [to] post enough with the specific language. - R2 Owner

#### Policies, incentives, and peer pressure (outer setting)

Our CFIR examination included the role of peer pressure, policies, and incentives. These were perceived by our post-test interviewees as not influencing their decision to make the changes. Awareness of the influence from policies and incentives on healthy eating interventions was low overall. In general, respondents had a similar initial negative reaction to the government’s role concerning restaurants. Most viewed government mandates as a burden on their workload and also viewed policies as restrictive and not beneficial for independently-owned restaurants. However, most respondents supported the idea of government incentives to promote healthy eating and support businesses, including, for example, wanting to see a collaborative relationship between government entities and restaurants to support the implementation of changes that could support healthier eating. For example, R2 owner mentioned that more recognition from government agencies toward independently-owned restaurants that support community health would be motivating.

Monetary incentives provided for their participation were seen as helpful, but the incentive was not seen as a key factor influencing the owner’s decision to participate. Despite communication of these benefits as part of the recruitment process, one owner cited not being aware of these, and wanting to participate given the perceived benefit for the restaurant. However, incentives were seen as important for staff engagement in the research process, as in the case of the stipend for the post-testing period interviews.

Regarding peer pressure, the owner from R1 and chefs at both restaurants noted being generally unaware of what other Latin American restaurants were doing to incorporate or promote HMI because they were too focused on their own restaurant and work. However, R2 owner noted that he tried to be aware of what other restaurants were doing to be competitive but stressed the importance of having his own vision to be unique as a business.

When you have a restaurant, you are aware of what other people are doing, and you want to be careful to be competitive, but also you want to be careful that you're not just following what others are doing because you're supposed to convey your own vision. - R2 Owner

## Discussion

This study examined the outcomes and determinants of tailored, restaurant-based interventions co-developed using HCD. The use of HCD was important in securing the buy in from key stakeholders, motivating changes that, while yielding mixed results, showcase the potential of this approach to create innovations in these complex settings that are often difficult to engage in public health interventions (37–39). This potential has been increasingly recognized in public health, including applications in global health and for chronic disease prevention (21). In the present study, the tailored interventions resulted in high acceptability, but were limited in influencing the sales of HMIs. While previous intervention research show the greater potential of combining increase in HMI with promotional efforts in community (non-chain) restaurants (13), these were not implemented jointly in the partner restaurants as the intensity and extent of the changes were limited to what the restaurant stakeholders were interested in and capable of achieving. The increase in HMI sales after the introduction of new, healthier items in R1 coincides with past research (40, 41), but arguably changes may have been larger if the intervention had incorporated increased promotion efforts, including those over social media. At the same time, the lack of significant changes in HMI in R2 coincide with mixed results found in past research examining the influence of promotional activities and healthier food sales alone (42, 43). While this part work has been mostly examining on-site promotional efforts, more research is needed to understand the influence and potential of social media for changing social norms and consumption patterns for restaurant choices. Social media research has documented the influence of this medium on children and adolescent food choices (44), but its use for the promotion of healthier foods is yet to be fully explored especially in restaurant settings.

This study contributes to such emerging area of work by applying implementation science to examine these interventions through a nuanced, theory-informed understanding of these results, demonstrating the benefits of expanding theoretical frameworks, as in the case of CFIR, to these complex, community-based contexts, with the potential for addressing persisting diet-related inequities.

The application of CFIR highlighted key influences within the interaction of the inner setting, individual characteristics, and the process used to develop the interventions. Our examination shows the importance of owner buy-in given the centralized decision making in the two participating independently-owned restaurants. The centralization is related to the relatively small size of the partner restaurants, with a small number of staff and the potential for high turn-over, where owners have to take on multiple roles, adding to the already high operational burden. This burden is compounded by perceived lack of staff capabilities to take on key roles (e.g., social media promotion, expansion of HMI), limiting the change intensity of the tailored interventions. This points to the need of addressing these structural issues as part of the intervention development process, to find ways to address time constraints and resource needs – aspects that tend to fall beyond the usual scope of public health interventions to promote healthier eating in restaurants.

Our findings concerning contextual or outer setting factors also merit further discussion. While our participants lacked awareness of policies or peer activities, these factors still have the potential to influence the restaurants. Their reactions to the questions about policies revealed that the majority of the owners and staff perceived existing regulations and interactions with public health entities (e.g., sanitation, city health department) as punitive, rather than supportive in connecting small business owners to resources and benefits. Both owners mentioned fines from the health department for lack of compliance with policies as their reason for mistrust in their relationship with government agencies. In the New York City context, where the partner restaurants are located, restaurants are subject to periodic, unannounced health inspections, potentially resulting in fines and a public downgrade in category (based on a letter system), further straining their relationship with the regulatory sector. Fines for lack of compliance with the health code, coupled with the cost of permits to run their businesses bring a financial burden for independently-owned restaurants. All of these findings demonstrate the need for government support for independently-owned Latin American restaurants and the need to assess ways to improve the relationship between restaurants and health-promoting agencies.

### Study strengths and limitations

We used objective measures to assess intervention outcomes, *via* sales data and the NEMS-R assessment. The use of CFIR provided a systematic way to examine the intervention determinants, guiding the design and analysis of the post-intervention interviews with restaurant stakeholders. Further, our interviews incorporated the perspective of multiple roles within the restaurants, by including front-of- the-house (e.g., servers) and back-of-the-house (e.g., chefs) staff in this exercise, an improvement from past research that tend to only examine owner and manager perspectives. Our use of a mixed-methods approach and multiple data sources allowed for data triangulation through different sources. For example, restaurant staff confirmed that the intervention did not increase their workload in both the staff rapid interviews conducted during site visits and from the post-testing in-depth interviews. Lastly, our joint examination of intervention outcomes and the implementation determinants provided a more in-depth analysis of the intervention. However, our study has limitations. We provided an in-depth analysis of two restaurants, with unique circumstances and tailored interventions that limit the generalizability of our findings to other restaurants. A second consideration is our approach to defining HMI, which was not based on a quantitative, nutrient-based analysis. We defined HMI through collaboration with experts, contextualizing the dishes within the restaurant menus and the potential for innovativeness, making the definition harder to replicate. We were unable to examine HMI on a *per capita* basis, as we could not capture individual consumption through the use of sales data. Our analysis of sales trends might have been influenced by sales fluctuations in response to COVID-19, which was a factor we were unable to capture in our analysis. Lastly, our examination of customer acceptance and satisfaction with the resulting intervention was limited to short intercept interviews during site visits, which did not capture a representative sample of customers and might be subject to social and selection bias.

### Conclusion and implications

The engagement of restaurants in healthy eating promotion interventions requires innovative ways to engage the sector and systematic approaches to examining the implementation of such interventions. The sector is difficult to reach and complex. Our emphasis was on Latin American restaurants, with insights that increase our understanding for working with other non-chain, independently-owned restaurants. While this study yielded mixed results in terms of HMI sales, the approach showed potential for augmenting owner buy-in and staff acceptance. More work is needed to facilitate innovative engagements and the application of implementation science to better understand the barriers and facilitators for intervention development, implementation, and sustainability in this sector. Future research should continue to engage the different levels of staff in these establishments to develop palatable changes that can sustain revenue while promoting healthier choices. More work is also needed beyond intervention development, expanding research to examine policy and regulatory level innovations to facilitate health promoting changes, especially within community, independently-owned restaurants, where the stakeholders engaged are typically within the communities these intervention aim to positively influence. Such work should also incorporate other aspects of the food systems influencing restaurant offerings, including ingredient costs and quality. The application of theoretical approaches from implementation science needs to be a part of these future efforts to both expand the current state of knowledge in food environment research while also expanding the application of theoretical models, as in the case of the CFIR, to new, more complex settings, continuing to build the field. The systematic and theory-driven approaches provided by implementation science can provide more focused approaches and learnings to develop interventions to best address persisting health inequities through community settings, as in the case of this work, engaging restaurants to address diet-related inequities among Latin American communities.

## Data availability statement

The datasets presented in this article are not readily available because given the nature of the data and in consideration to our partner restaurants, we are unable to share sales and qualitative data. The authors will consider reasonable data requests, but sharing will be limited to raw, anonymized data. Requests to access the datasets should be directed to MF, mfuster@tulane.edu.

## Ethics statement

The studies involving human participants were reviewed and approved by Tulane University Institutional Review Board. Written informed consent for participation was not required for this study in accordance with the national legislation and the institutional requirements.

## Author contributions

MF, ED, MH, DR, CS, MK, BE, and TH contributed to the writing and editing of the manuscript. MF is the principal investigator and primary author, secured project funding, supervised data collection and analysis, and led the writing of the manuscript. ED co-led the interview efforts, contributed to the data analysis, and assisted in the writing and revision process. CC co-led the interview efforts, carried out the site visits, led the collection of sales data and social media monitoring, and contributed to the analysis. CS advised and contributed to the analysis of the sales data. MK assisted in the qualitative analysis write-up and revisions of the manuscript. DR, BE, MH, and TH advised on all aspects of the manuscript, study conceptualization, and contributed to the write-up and revisions of the manuscript. All authors contributed to the article and approved the submitted version.

## Funding

The research was supported by the NIH-National Heart, Lung, and Blood Institute (Award # K01HL147882). Additional funding support for TH was provided by the Centers for Disease Control and Prevention (U48DP006396). The funders had no role in the design, analysis, or writing of this article.

## Conflict of interest

The authors declare that the research was conducted in the absence of any commercial or financial relationships that could be construed as a potential conflict of interest.

## Publisher’s note

All claims expressed in this article are solely those of the authors and do not necessarily represent those of their affiliated organizations, or those of the publisher, the editors and the reviewers. Any product that may be evaluated in this article, or claim that may be made by its manufacturer, is not guaranteed or endorsed by the publisher.
