# Supplementary material for: Evaluating the outcomes and implementation determinants of interventions co-developed using human-centered design to promote healthy eating in restaurants: an application of the consolidated framework for implementation research
Source: Front Public Health. 2023 May 18;11:1150790. doi: 10.3389/fpubh.2023.1150790 (PMC10233011; doi:10.3389/fpubh.2023.1150790)
Supplement: Supplementary file 1 [file Table_1.DOCX]

Supplementary Table 1. Changes in Daily Healthy Menu Items (HMI) sold

|  | R1: New HMIs and Menu Redesign (n=216) | | | | R2: Social media promotion of HMIs  (n=185) | | | |
| --- | --- | --- | --- | --- | --- | --- | --- | --- |
|  | Coef. | Std. Err. | t | p-value | Coef. | Std. Err. | t | p-value |
| Time since start of study | 0.004 | 0.01 | 0.41 | 0.42 | 0.04 | 0.04 | 1.06 | 0.29 |
| Break in trend upon intervention start | 30.60 | 5.51 | 5.55 | <0.001 | -0.24 | 2.60 | -0.09 | 0.93 |
| Change in daily trend after intervention start | -0.22 | 0.09 | -4.48 | 0.014 | -0.10 | 0.05 | -2.02 | 0.04 |
| Break in trend upon intervention end | -2.78 | 2.35 | -1.18 | 0.239 | -1.52 | 2.45 | -0.62 | 0.54 |
| Change in daily trend after intervention end | 0.20 | 0.09 | 2.23 | 0.027 | 0.02 | 0.05 | 0.44 | 0.66 |
| Constant | 1.61 | 0.44 | 3.67 | <0.001 | 13.90 | 1.56 | 8.89 | <0.001 |
